# Kaempferol attenuated diabetic nephropathy by reducing apoptosis and promoting autophagy through AMPK/mTOR pathways

**DOI:** 10.3389/fmed.2022.986825

**Published:** 2022-11-30

**Authors:** Hongqin Sheng, Duo Zhang, Jiaqi Zhang, Yanmei Zhang, Zhaoyu Lu, Wei Mao, Xusheng Liu, Lei Zhang

**Affiliations:** ^1^State Key Laboratory of Dampness Syndrome of Chinese Medicine, The Second Affiliated Hospital of Guangzhou University of Chinese Medicine, Guangzhou, China; ^2^The Second Clinical Medical College of Guangzhou University of Chinese Medicine, Guangzhou, China; ^3^Guangdong Provincial Key Laboratory of Clinical Research on Traditional Chinese Medicine Syndrome, Guangzhou, China

**Keywords:** diabetic nephropathy, Kaempferol, apoptosis, autophagy, AMPK/mTOR pathway

## Abstract

**Introduction:**

Renal podocyte injury, apoptosis and autophagy are involved in the occurrence and development of diabetic nephropathy (DN). Kaempferol (KPF) has the promotion of autophagy and inhibition of apoptosis properties in the development of miscellaneous diseases, but these functions in DN have not yet been elucidated.

**Methods:**

We used *db/db* mice to evaluate the protective role of KPF on DN. The anti-DN effect of KPF was evaluated by urine albumin-to-creatinine ratio and pathological changes of kidney tissue. Injury of podocytes was observed through Transmission electron microscopy. Immunofluorescence, Western blot, and Immunohistochemistry were used to detect the protein expression of podocyte-associated molecules, autophagy, and AMPK/mTOR pathway.

**Results:**

We demonstrated that KPF treatment significantly attenuated diabetes-induced albuminuria and glycolipid metabolism dysfunction. In addition, KPF alleviated mesangial matrix expansion, glomerular basement membrane thickening and loss or fusion of podocytes. Mechanistically, KPF treatment regulated the expression of autophagic proteins (upregulated LC3II, Beclin-1, Atg7 and Atg 5, and downregulated p62/SQSTM1), accompanied by inhibited renal apoptosis (downregulated Caspase 3 and Bax, and upregulated Bcl-2). KPF could significantly regulate the AMPK/mTOR signaling pathways by increasing p-AMPK and decreasing p-mTOR expressions.

**Discussion:**

In conclusion, KPF might have a protective effect on DN through reduced apoptosis and enhanced podocytes autophagy, which were correlated with regulating AMPK/mTOR pathways.

## Introduction

Diabetic nephropathy (DN) is a serious diabetic angiopathy, manifesting as microvascular complications, and a leading cause of end-stage renal disease (1). In the kidney glomerulus, podocytes are pivotal in maintaining glomerular filtration barrier function. The loss and dysfunction of podocytes are closely associated with proteinuria, the hallmark of early renal injury in DN (2), and contribute to aggravated glomerular injury and the progression of DN (3, 4).

Autophagy is the way of refreshing newer, healthier cells through catabolizing damaged cells. It is an essential self-repair mechanism in maintaining cell homeostasis (5). Podocytes show high rates of autophagy under normal conditions. Studies have confirmed that reduced podocytes autophagy induces podocyte function alteration that may exacerbate diabetic renal damage (6, 7). Most of the research in podocyte autophagy has focused on the mammalian target of rapamycin (mTOR) and AMP-activated protein kinase (AMPK) signaling pathways, which play an important role in accelerated podocyte injury in DN (8–11). AMPK is a key regulator of energy homeostasis and a vital energy sensor that can promote autophagy. On the other hand, mTOR can inhibit autophagy, which is a significant regulator of cellular and organismal growth (12). High glucose conditions can inhibit the AMPK signaling pathway and promote mTOR activation, thus inhibiting podocyte autophagy and aggravating kidney injury (13). Thus, autophagy regulation should be a therapeutic target to prevent DN. Agents that can regulate the activity of mTOR and AMPK may have a therapeutic effect on DN.

Kaempferol (KPF), which could be extracted from tea leaves, broccoli, hazelnuts, propolis, grapefruit and other green plants, belongs to the family of natural flavanol. It has been reported for its roles as a potential anti-diabetic, anti-obesity and anti-fibrosis agent, attributed to its potent anti-inflammatory, anti-oxidative, and anti-atherosclerotic effects (14–16). It has been shown in studies that KPF could promote autophagy and inhibit apoptosis *via* the regulation of AMPK/mTOR pathway in several conditions, including pancreatic β-cell dysfunction (17, 18), memory deficits (19), cerebral ischemic-reperfusion injury (20), hepatic cancer (21), and HeLa cells (22). The nephroprotective effect of KPF on streptozotocin induced type-1 DN mice has been reported in previous studies, due to its effect of ameliorating inflammation, which correlates with decreased TRAF6 levels (23), the upregulation of the Nrf-2/HO-1 axis (24), the enhanced GLP-1 and insulin release, and the inhibition of RhoA/Rho kinase activity (25). However, the renal protective effects of KPF on a type-2 DN model of *db/db* mice remain unclear. In addition, studies on whether KPF could prevent the progression of DN through regulating autophagy *via* AMPK/mTOR pathway are lacking.

Consequently, in our current study, we aim to investigate whether KPF treatment has therapeutic effects on type-2 DN model of *db/db* mice and to examine the mechanism underlying the protective effects of KPF on DN by focusing on the regulation of podocyte autophagy *via* AMPK/mTOR pathway.

## Materials and methods

### Reagents and chemicals

Kaempferol was purchased from Sigma-Aldrich (St. Louis, MO, USA) and was suspended in 1% sodium carboxymethyl cellulose (CMC-Na). The antibodies against SQSTM1/p62 (23214), Beclin1 (3738), LC3II (83506), Bcl-2 (3498), Bax (14796), Caspase3 (9662), Atg7 (8558), AMPK (5832), p-AMPK (50081), mTOR (2983), p-mTOR (5536, 2976), Horseradish Peroxidase (HRP)-linked anti-rabbit Immunoglobulin G (IgG) (7074), HRP-linked anti-mouse IgG (7076) antibodies, and DAB Substrate Kit were all purchased from Cell Signaling Technology. Atg5 (ab108327), the antibodies against Nephrin (ab216341) and Wilms’ tumor protein-1 (WT1) (ab89901) were purchased from Abcam. The antibody against β-actin (BM0627) and terminal deoxynucleotidyl transferase dUTP nick end labeling (TUNEL) apoptosis detection Kit (MK1018) were from Boster Biological Technology. Enhanced chemiluminescence (ECL) reagent was obtained from Millipore.

### Experimental animals

Eight-week-old male C57BLKS/J *db/db* (*n* = 15) and *db/m* mice (*n* = 5) were obtained from the Model Animal Research Center of Nanjing University (experimental animal license No. 32002100006993). All mice were housed in the SPF level animal room, where the temperature was controlled at 23 ± 3^°^C and humidity at 55 ± 15% with a 12 h light and dark cycle and fed with a standard rodent diet and sterile water. After 1 week of acclimatization, the *db/db* mice were randomly divided into 3 groups (*n* = 5 each) *db/db*, *db/db* + Low Kaempferol (LKPF, Kaempferol 50 mg/kg/day dissolved in 1% CMC-Na) and *db/db* + High Kaempferol (HKPF, Kaempferol 100 mg/kg/day dissolved in 1% CMC-Na). KPF was administered through oral gavage, and the dose of KPF is referred to the previous study (26). The *db/m* mice were given the same daily volume of 1% CMC-Na solution. After 12 weeks of KPF treatment, all mice were sacrificed for the experiment after fasting overnight. The experimental procedures used in the present study were approved by the Animal Care and Ethics Committee of Guangzhou University of Chinese Medicine (ethical batch number: 2018076) and were in accordance with the internationally accepted principles for laboratory animal use and care.

### Urine and serum measurement

All mice were weighed every week, and fasting plasma glucose (FPG) was tested every 2 weeks. We collected urine samples using metabolic cages every 4 weeks. Urine albumin-to-creatinine ratio (UACR) was measured using an automatic analyzer. The mice were sacrificed after KPF treatment for 12 weeks. Blood was sampled from eyeball blood collection. Serum creatinine (SCr), total cholesterol (TC), and low-density lipoprotein cholesterol (LDL-C) were detected using an automatic analyzer by the clinical laboratory of Guangdong Provincial Hospital of Chinese medicine. At the time of sacrifice, renal tissues were collected. Kidney tissues were either fixed in 4% paraformaldehyde for histological analysis or fast-frozen in liquid nitrogen and stored at –80°C for protein expression detection.

### Histological analysis

The kidney tissues were fixed in 10% formalin buffer for 48 h and then embedded in paraffin, which were cut into 5-μm thick, and stained with periodic acid–Schiff (PAS) and Hematoxylin and Eosin (H&E) to determine the mesangial matrix expansion of glomerulus and to estimate the kidney damage. PAS and H&E-stained sections were observed under an Olympus IX71 microscope.

### Immunohistochemistry

For Immunohistochemistry (IHC), 5-μm thick and paraffin-embedded kidney sections were separately stained with primary antibodies against nephrin (1:1000), Bcl-2 (1:500), Bax (1:500), Caspase3 (1:500), Atg5 (1:100), p-AMPK (1:100), and p-mTOR (1:100) at 4°C overnight. Next, incubated by the secondary antibody and 3,3′3′-Diaminobenzidine (DAB) kit, the sections were viewed under an Olympus IX71 microscope.

### Immunofluorescence

Immunofluorescence (IF) assay was used to assess the expression of WT1, LC3II and nephrin protein in kidney tissues. A total of 0.25% Triton X-100 was used to permeabilize 5-μm thick kidney sections for 15 min, and 5% Bovine Serum Albumin (BSA) was used to block them for 1 h at room temperature. Then, the sections were stained with primary antibodies against WT1 (1:200), LC3II (1:200), and nephrin (1:500) overnight at 4°C, followed by secondary Cy3-labeled goat anti-rabbit IgG (A0516, Beyotime), Cy3-labeled goat anti-mouse IgG (A0521, Beyotime) or FITC-labeled goat anti-rabbit IgG (A0561, Beyotime). The cell nuclei were labeled with 4′,6-diamidino-2-phenylindole (DAPI), and images were obtained with fluorescence microscopy.

### Terminal deoxynucleotidyl transferase dUTP nick end labeling (TUNEL) assay

The kidney sections were dewaxed and hydrated in graded alcohol series and were incubated with Proteinase K (1:200 diluted by 0.01 M Tris/Hcl, pH 7.5) for 15 min at 37°C to enhance tissue permeability. After rinsing 3 times with 0.01 M TBS, each section was added 20 μl labeling buffer to keep moist. The labeling buffer was composed of 1 μl Terminal deoxyribonuclease transferase (TDT), 1 μl Biotin-labeled DUTP (Bio-DUTP), and 18 μl buffer solution. After incubating in a humidified environment for 2 h at 37°C protecting from light, the sections were rinsed for 3 times, and each section was added 5% serum albumin fraction for blocking 30 min at room temperature. And then, each section was added SABC-FITC (1:200 diluted) for 30 min at 37°C. Rinsing 4 times with 0.01 M TBS, At last the cell nuclei were stained with DAPI, and images were obtained with the fluorescence microscopy.

### Transmission electron microscopy

We used transmission electron microscopy (TEM) to evaluate ultrastructural. Kidney tissues were sliced into cubes and in fixation in 2.5% glutaraldehyde. Samples were handled in Wuhan service biotechnology and detected by transmission electron microscope (JEM-1400 PLUS, Tokyo, Japan).

### Western blot analysis

Renal tissues were lysed in Radioimmunoprecipitation assay buffer (RIPA) buffer. A total of 30 μg per well protein were separated on 10 or 15% sodium dodecyl sulfate-polyacrylamide gel electrophoresis (SDS-PAGE) and subsequently immunoblotted on Polyvinylidene fluoride or polyvinylidene difluoride (PVDF) membranes. Densitometric quantification of the protein bands was analyzed by Image Lab System (Bio-Rad Laboratories, Inc.). The primary antibodies are listed as follows: nephrin, SQSTM1/p62, Beclin1, LC3II, Bcl-2, Bax, Caspase 3, Atg7, AMPK, p-AMPK, mTOR, p-mTOR, β-actin 1:1,000.

### Statistical analysis

All results were expressed as the mean ± standard deviation (SD). Statistical analysis of all data was performed by using Graphpad Prism 7.0 software (San Diego, CA, USA). Statistical significance was determined using a one-way analysis of variance (ANOVA) in multiple comparisons. A value of *p* < 0.05 was considered to be statistically significant.

## Results

### Kaempferol decreased urine albumin-to-creatinine ratio and improved glycolipid metabolism dysfunction of *db/db* mice

**FIGURE 1:**
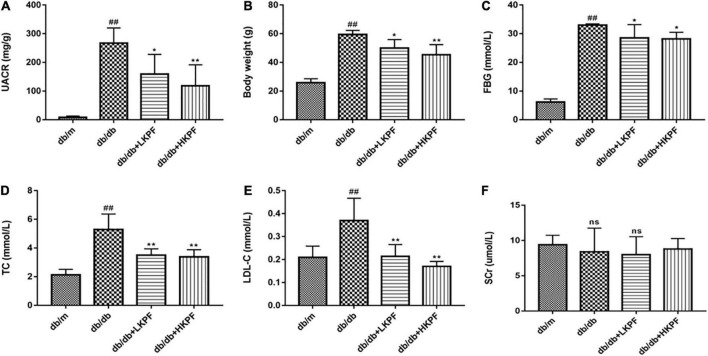
Kaempferol alleviated dysregulated UACR, BW, FBG and dyslipidemia levels of *db/db* mice. **(A)** Urine albumin-creatine ratio (UACR) (mg/g), **(B)** Body weight (g), **(C)** Fasting blood glucose (FBG) (mmol/L), **(D)** Total cholesterol (TC) (mmol/L), **(E)** Low density lipoprotein-cholesterol (LDL-C) (mmol/L), and **(F)** serum creatinine (SCr) (μmol/L) were measured after KPF treatment. The data were presented as the mean ± SD (*n* = 5, *^##^p* < 0.01 vs. *db/m* mice; *p < 0.05 and **p < 0.01 vs. *db/db* mice; ns, not significant).

In comparison with *db/m* mice, *db/db* mice had a significantly higher level of urine albumin-creatine ratio (UACR), body weight (BW), fasting blood glucose (FBG), TC, and low-density lipoprotein-cholesterol (LDL-C). A dose-dependent reduction in these indicators was observed in *db/db* mice after administration of KPF (either 50 or 100 mg/kg/day) for 12 weeks (Figure 1).

### Kaempferol ameliorated renal histological damage in *db/db* mice

**FIGURE 2:**
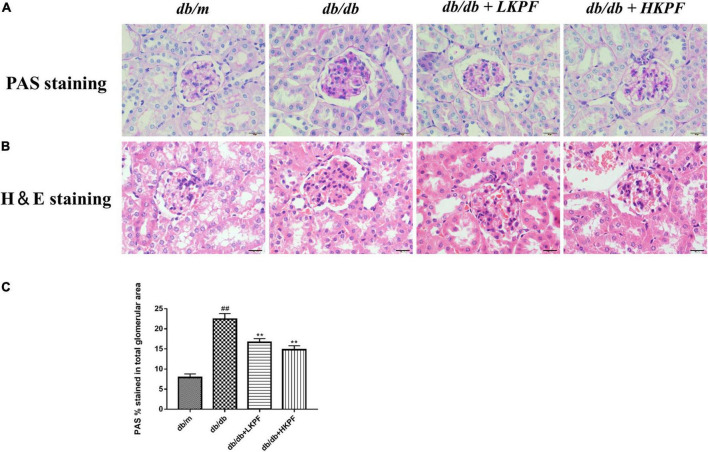
Kaempferol ameliorated renal histological damage in *db/db* mice (400×, bar = 20 μm). **(A)** Kaempferol treatment ameliorated glomerular mesangial matrix deposition in *db/db* mice (PAS staining). **(B)** Kaempferol treatment ameliorated morphology in the kidneys of *db/db* mice (H&E staining). **(C)** Quantification of PAS stained (the glomerular mesangial matrix area) for each group. The data were presented as the mean ± SD (*n* = 5, *^##^p* < 0.01 vs. *db/m* mice; **p < 0.01 vs. *db/db* mice). H&E, Hematoxylin and Eosin; PAS, Periodic Acid–Schiff.

The morphological changes of the kidney were observed using PAS and H&E staining. Compared with *db/m* mice, the kidneys of *db/db* mice showed mesangial matrix expansion and extracellular matrix deposition. Treatment with KPF for 12 weeks significantly reduced the accumulation of extracellular matrix and the expansion of renal mesangial in *db/db* mice (Figure 2).

### Kaempferol attenuated podocytes injury in *db/db* mice

**FIGURE 3:**
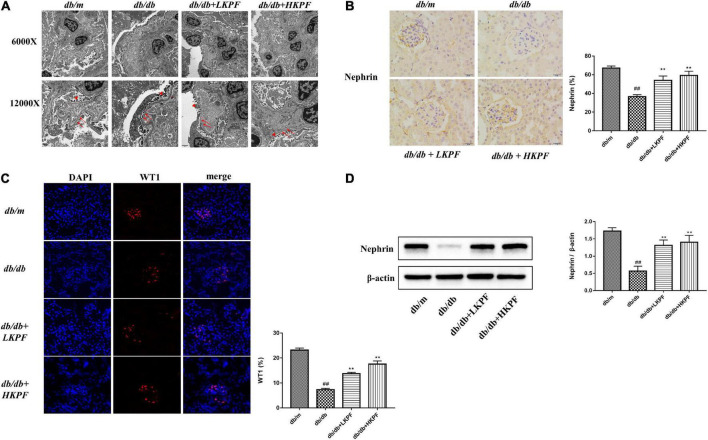
Kaempferol attenuated podocytes injury in *db/db* mice. **(A)** TEM (6000×, bar = 2 μm and 12000×, bar = 1 μm) micrograph, showing the GBM thickness (arrows) and FPs width (triangle). **(B)** Immunohistochemical (IHC) analysis of nephrin positive cells (400×, bar = 20 μm) and quantification of IHC staining for nephrin (*n* = 5). **(C)** IF analysis of podocyte nucleoprotein WT1 in kidney tissue. The tissues were stained with an antibody against WT1 (red), DAPI (blue) was used to stain the cellular nucleus (400×, *n* = 5). And quantification of IF stained for WT1 (*n* = 5). **(D)** WB assay and quantitative analysis of nephrin expression after KPF treatment, β-actin was used as internal references; data from each group are expressed as the mean ± SD (*n* = 3) from three repeated WB experiments. The data were presented as the mean ± SD (*^##^p* < 0.01 vs. *db/m* mice; **p < 0.01 vs. *db/db* mice). TEM, Transmission electron microscopy; IHC, Immunohistochemical; WB, Western blot; GBM, glomerular basement membrane; FPs, foot processes.

Transmission electron microscopy revealed that podocytes were damaged with foot processes obvious loss and fusion, and the glomerular basement membrane thickening in *db/db* mice compared to *db/m* mice. However, these injuries were alleviated with KPF treatment (Figure 3A). Then, Western blot (WB) and IHC analysis were conducted to determine nephrin expression levels, while Immunofluorescence (IF) analysis determine WT1 expression levels. The levels of these two podocyte-specific proteins (nephrin and WT1) were significantly reduced in glomeruli of *db/db* mice relative to *db/m* mice, which was significantly ameliorated with KPF treatment (Figures 3B–D). These results indicated that KPF exerts a podocyte protection effect in a mice model of DN.

### Kaempferol reduced apoptosis in the kidneys of *db/db* mice

**FIGURE 4:**
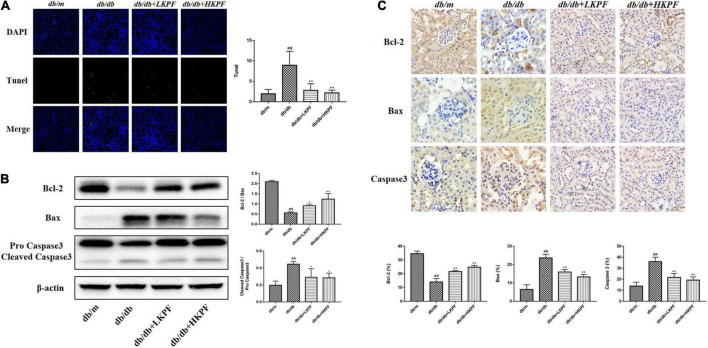
Kaempferol reduced apoptosis in the kidneys of *db/db* mice. **(A)** TUNEL-positive cells (green) were stained in glomeruli after Kaempferol treatment, DAPI (blue) was used to stain the cellular nucleus (400×, *n* = 3). **(B)** WB assay and quantitative analysis of apoptosis-related proteins Bcl-2, Bax, and Caspase 3 expressions after Kaempferol treatment, β-actin was used as internal references; data from each group were expressed as the mean ± SD (*n* = 3) from three repeated WB experiments. **(C)** IHC assay and quantitative analysis of apoptosis-related proteins: Bcl-2, Bax, and Caspase 3 (400×, n = 5). (*^##^p* < 0.01 vs. *db/m* mice; *p < 0.05 and **p < 0.01 vs. *db/db* mice). IHC, Immunohistochemical; WB, Western blot.

TUNEL staining was used to assess cell apoptosis in the kidneys of *db/db* mice. Our results indicated that the number of TUNEL-positive cells significantly increased in the kidneys of *db/db* mice, whereas KPF treatment reduced kidney cell apoptosis (Figure 4A). To further determine the effect of KPF on apoptosis in the kidneys of *db/db* mice, the apoptosis-related proteins were assessed by WB. The pro-apoptotic proteins Bax and Caspase 3 were upregulated and the anti-apoptotic protein Bcl-2 was downregulated in *db/db* mice in comparison to *db/m* mice, which were reversed by KPF treatment (Figure 4B). Moreover, the results of IHC analysis were consistent with the WB results (Figure 4C), which revealed that the apoptosis process was increased in the kidneys of *db/db* mice and decreased by KPF treatment.

### Kaempferol enhanced autophagy in podocytes and regulated AMPK/mTOR pathways in the kidneys of *db/db* mice

**FIGURE 5:**
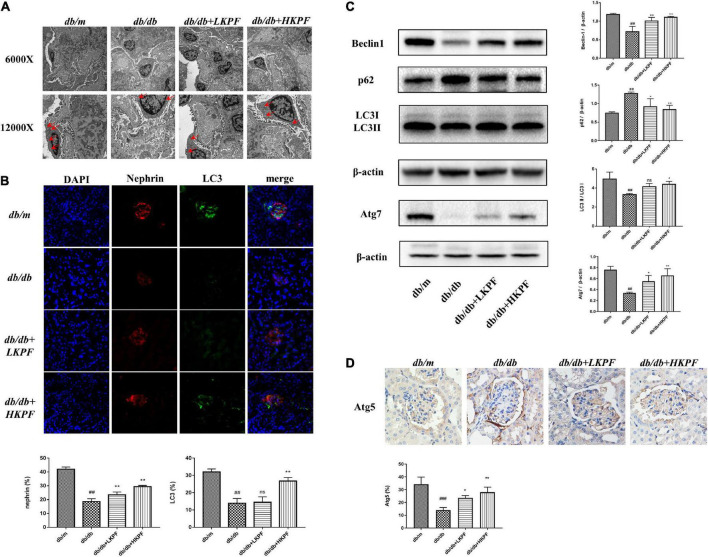
Kaempferol enhanced autophagy in podocytes in the kidneys of *db/db* mice. **(A)** Autophagosome formation was detected in kidneys by using TEM (6000×, bar = 2 μm and 12000×, bar = 1 μm), red arrows indicated autophagosomes. **(B)** IF analysis of the autophagosome marker LC3II and podocyte marker protein nephrin in kidney tissue. The tissues were stained with antibodies against LC3II (green) and nephrin (red). DAPI (blue) was used to stain the cellular nucleus (400×, *n* = 5). **(C)** WB assay and quantitative analysis of p62, Beclin1, LC3, and Atg7 expressions after Kaempferol treatment, β-actin was used as internal references; data from each group are expressed as the mean ± SD (*n* = 3) from three repeated WB experiments. **(D)** IHC assay and quantitative analysis of apoptosis-related protein: Atg5 (400×, *n* = 3). ^#^*p* < 0.05, ^##^*p* < 0.01 and ^###^*p* < 0.001 vs. *db/m* mice; *p < 0.05 and **p < 0.01 vs. *db/db* mice; ns, not significant). TEM, Transmission electron microscopy; IF, Immunofluorescence; WB, Western blot.

**FIGURE 6:**
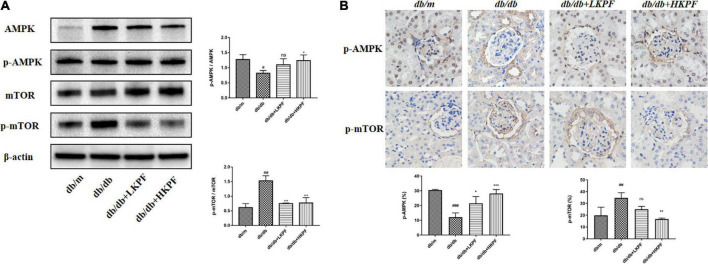
Kaempferol regulated AMPK/mTOR pathways in the kidneys of *db/db* mice. **(A)** WB assay and quantitative analysis of AMPK, p-AMPK, mTOR, and p-mTOR expressions after Kaempferol treatment, β-actin was used as internal references; data from each group are expressed as the mean ± SD (*n* = 3) from three repeated WB experiments. **(B)** IHC assay and quantitative analysis of p-AMPK and p-mTOR expressions (400×, *n* = 3). (*^##^p* < 0.01 and *^###^p* < 0.001 vs. *db/m* mice; *p < 0.05, **p < 0.01, and ***p < 0.001 vs. *db/db* mice; ns, not significant). IHC, Immunohistochemical; WB, Western blot.

Transmission electron microscopy was used to detect the presence of autophagosomes, as shown by the red arrowheads in Figure 5A. The results revealed a decreased number of autophagosomes in podocytes in *db/db* mice compared with *db/m* mice, which was elevated with KPF treatment (Figure 5A). We also assessed the expression of LC3II in podocytes by IF, which was decreased in *db/db* mice in comparison with *db/m* mice and was reversed by KPF treatment (Figure 5B). The WB analysis of autophagy-related proteins (LC3II, p62, Beclin1, and Atg7) and IHC analysis of Atg5 supported the above results (Figures 5C,D). Furthermore, we assessed the protein expression of AMPK, mTOR and their phosphorylation protein both by WB and IHC. Compared with *db/m* mice, the expression of mTOR phosphorylation (p-mTOR) was increased and the expression of AMPK phosphorylation (p-AMPK) was decreased in *db/db* mice, which were reversed by KPF treatment (Figures 6A,B). These data indicated that KPF could enhance autophagy in podocytes and regulate AMPK/mTOR pathways in DN.

## Discussion

This study demonstrated that Kaempferol (KPF) could significantly reduce UACR, improve glycolipid metabolism dysfunction, as well as reduce renal histopathological damage, especially podocytes injury of *db/db* mice *via* reducing apoptosis and promoting podocytes autophagy, which is correlated with the regulation of AMPK/mTOR pathways.

Proteinuria is the hallmark of DN and has been widely regarded as an independent risk factor for renal accelerated damage, which is caused by the damage of glomerular filtration barrier, including renal mesangial expansion, extracellular matrix accumulation, especially podocyte injury (27, 28). Podocyte dysfunction has been considered a breakthrough in deciphering the molecular mechanisms of DN (29).

Nephrin and WT1 have been used as biomarkers for the evaluation of podocyte damage. Our data showed decreased expressions of nephrin and WT1, accompanied by foot processes fusion and glomerular basement membrane thickening in the kidneys of *db/db* mice, which were significantly ameliorated with KPF treatment, showing the protective effect of KPF on podocyte in DN. This finding in our study was consistent with the previous findings by Yuanping Li et al. (30), and Xinyu Wang et al. (31), which showed the inhibition effect of KPF on podocyte apoptosis under hyperglycemic conditions *in vitro*. Impaired autophagy mediated imbalance of homeostasis in podocytes, contributing to podocyte dysfunction and further renal injury in DN (32). In the present study, indicators of autophagy such as light chain 3 (LC3), p62 (SQSTM1/sequestosome 1), Beclin-1, Atg5, and Atg7 were assessed. LC3 is now widely regarded as a monitor of autophagy activity. The detection of LC3 conversion (LC3-I to LC3-II) is the most reliable approach to evaluate autophagy activity (33). An alternative approach to detect autophagic flux is to measure the degradation of p62 (34). As a selective autophagy substrate, p62 can bind LC3, which indicates its significance in monitoring autophagy suppression (35). As one of the first autophagy effectors identified in mammals (36), Beclin-1 could interact with PtdIns (3)-kinase (Vps34) and initiate the nucleation step of autophagy to begin autophagic flux and also participates in following steps involving the fusion of autophagosomes to lysosomes (37). Atg5 and Atg7 are both essential for the formation of microtubule-associated protein 1 light chain 3-phosphatidylethanolamine (MAP1LC3-PE) conjugation and autophagosome formation (38, 39), mediating membrane expansion and maturation of autophagosomes. In our findings, KPF treatment increased the numbers of autophagosomes in podocytes, the double-membrane vesicles newly formed during autophagy. In addition, KPF effectively promoted the expression of LC3II in podocytes, with an increased expression of Beclin-1, Atg5, Atg7 and a decreased expression of p62 in the kidneys of *db/db* mice, suggesting that the KPF could prevent DN *via* regulating podocyte autophagy, which has not been reported by previous studies.

A multitude of signaling molecules is involved in podocyte autophagy, including mTOR and AMPK (40). The inhibitory function of mTOR might be linked to nutrient signals to regulation of autophagy (41). The hyperactivation of the mTOR pathway in DN is crucial to the injury of podocytes (42). AMPK could maintain energy homeostasis by serving as a cellular energy status sensor (43). AMPK has been implicated in autophagy induction in response to glucose starvation (44). Promoting AMPK activation could protect podocytes from high glucose-induced injury (45). Our results revealed the upregulation of AMPK phosphorylation as well as the downregulation of mTOR phosphorylation in KPF treated *db/db* mice. Overall, we demonstrated that KPF treatment alleviated podocyte autophagy dysfunction in *db/db* mice.

Collectively, our findings indicate the renal protective effect of KPF on type 2 DN *db/db* mice that KPF treatment reduced apoptosis and enhanced podocytes autophagy through AMPK/mTOR pathways. However, the exact mechanism of how KPF regulated AMPK/mTOR pathway remains to be investigated.

## Conclusion

In summary, our study demonstrated that KPF could decrease UACR, improve glycolipid metabolism dysfunction and podocytes injury of *db/db* mice. Its protective effect might be attributed to reducing apoptosis and enhancing podocytes autophagy, which are correlated with regulating AMPK/mTOR pathways. Our findings may be implicated in the potential therapeutic role of KPF in future DN treatment. Further research on how KPF regulated AMPK/mTOR pathway is warranted.

## Data availability statement

The original contributions presented in this study are included in the article/supplementary material, further inquiries can be directed to the corresponding author.

## Ethics statement

The animal study was reviewed and approved by the Animal Care and Ethics Committee of Guangzhou University of Chinese Medicine.

## Author contributions

LZ acquired funding for the research, contributed to the conception and design of the experiments, and analysis and interpretation of the data. HS drafted the manuscript. HS and DZ contributed to the performance of the experiments and the acquisition and analysis of data. DZ, JZ, YZ, and ZL contributed to the performance of some experiments and analysis of data. WM contributed to providing experimental suggestions. XL contributed to the design of the experiment and edited this manuscript. All authors reviewed the manuscript and approved the final version to be published.
